# Metallic *Versus* the Plastic Base for Artificial Dentures

**Published:** 1894-10

**Authors:** I. Norman Broomell

**Affiliations:** Philadelphia


### METALLIC *VERSUS* THE PLASTIC BASE FOR ARTIFICIAL DENTURES.[1]

BY I. NORMAN BROOMELL, D.D.S., PHILADELPHIA.

WHILE the reputation of the dental profession in general has been gradually advancing in regard to their knowledge of therapeutics, and in the treatment of oral diseases, together with a still greater advancement in all operations upon the natural teeth, there has not been a corresponding progress in prosthetic dentistry. The existence of this condition will hardly be denied, therefore the acceptance of its truth naturally leads us to inquire into the causes producing it.

Numerous reasons might be assigned for this rather unfortunate circumstance, but to my mind no one cause has been so instrumental in producing the result as the introduction of the plastic or vegetable compounds as a base for artificial teeth. It is very true that these have their place in dentistry, and circumstances frequently occur when it would appear almost impossible to succeed without them; but it is not the use so much as the abuse of these materials that has been working the damage. First, as to their use. Celluloid and zylonite having proved themselves insufficiently durable, my remarks shall have no reference to them, and I shall consider vulcanite rubber as the only plastic base now in use. What are the circumstances under which rubber can be used to best advantage? Undoubtedly in full dentures, when great absorption has taken place, and something of a plastic nature is required to give sufficient bulk to the denture, to re-establish the proper contour and fulness.

It would be difficult to find a better material from which to construct an interdental splint than vulcanite rubber, while in full metal dentures its use as the means of attachment is invaluable. Under these few conditions rubber may be used to advantage.

Now as to its misuse and the ill results accruing therefrom. What are some of these ill results? *First*, a lack of any thought or care for prosthetic dentistry by the best members of the profession, with a consequent decline of this branch of our calling, and a desire to separate from it. *Second*, the advancement of quackery. *Third*, a desire upon the part of those persons who place a greater value upon the absence of pain than they do the presence of their natural teeth, preferring to part with these precious organs and

---

[1] Read before the Odontological Society of Pennsylvania, April 14, 1894.

substitute artificial ones. *Fourth*, an increase in diseased conditions of the mouth, both of the soft tissues and of the teeth themselves.

The first ill result is certainly one to be deplored, much harm frequently coming from the growing desire of many members of the profession to shun mechanical work. To my mind the perfect dentist, the dentist most able to comprehend the wants and needs of his patients, must give equal attention to each branch of his calling, be it operative, therapeutic, or prosthetic. To do this it is not necessary that he should actually perform all these duties himself, but his mind should be instrumental in controlling them, permitting no prejudice to make him give all thought and attention to operative work, with an utter disregard as to what may be the ill results of his inattention to prosthetic work.

By way of illustration permit me to report a recent case in my own practice. The patient, a lady, had for a number of years been under the care of a prominent dentist in another city. Upon examining her mouth I found a number of perfectly-inserted gold fillings, plainly showing that the operator possessed much skill in this direction. I also found the upper bicuspids and molars on the left side as well as one or two teeth upon the right side to be missing. For a number of years the lady had urged her dentist to construct a plate for her, or in some way close up the deficiency. This he refused to do, saying that she could not wear a plate on account of a bony ridge in the hard palate. Every time she called upon him she made the same request, and always received the same negative reply. The experienced dentist may well know what had taken place by the time she came into my hands. Existing as there did a marked disposition to pyorrhœa alveolaris, the lower teeth from the want of occlusion had left their sockets, and most of them were striking the gum in the upper jaw, thus excluding all possibility of remedying the trouble without extracting the lower teeth. And yet this practitioner, wedded, as he must have been, to operative dentistry, stood idly by and permitted these teeth to be lost, or worse than lost, as they were rendered absolutely useless, simply because he could not exert himself sufficiently to prevent it. What excuse could be offered for such gross neglect of duty. Certainly the ridge in the hard palate was not sufficient to bar all possibility of inserting a partial denture, and even admitting that it was, a small clasp plate could have been constructed, and it would have been many years before the clasps would have caused the utter destruction of so many teeth, and it is doubtful if they would ever have done so. The conclusions to be drawn from this case are these. Had vulcanite never been introduced into the dental laboratory, it is safe to assume that this "operator" would have had a better schooling in metal plate-work, and having this extra knowledge, he would have used it to advantage by constructing a partial denture for the patient, and thereby preserved the teeth which she is now compelled to sacrifice.

The second ill result, the advancement of quackery. The standing of a profession is known by the acts of its members. Who are the members of the dental profession? It is to be feared that the public look upon all dentists as members of it. Probably in a measure they are. All quacks may be dentists, but fortunately all dentists are not quacks. Vulcanite rubber is the nucleus from which sprang dental quackery. Can we for a moment suppose that this class of dentists would exist in such great numbers if they were compelled to form all their dentures upon a metal base?

The third ill result, a desire upon the part of those persons who place a greater value upon the absence of pain than they do upon the presence of the natural teeth to part with these precious organs and substitute artificial ones. How frequently do we hear our patients remark that they intend to have all their teeth extracted to make room for a lifeless substitute. Would such thoughts occur to them if they were compelled to pay the price of a gold plate to accomplish their desires? These people imagine they would be loosing nothing and gaining everything. How frequently and how cruelly are they disappointed. Think of the changed expression, the lost lines of nature's beauty, the impaired articulation ; with all these lost they have probably gained the coveted absence of pain, but the price has been the loss of that which, as God's creatures, we are given and bound to preserve, nature's own cheerful countenance.

For the sake of argument we will imagine the majority of all our patients to be poor, and the existence of this unfortunate condition promotes the idea of economical dentistry. Naturally enough they look upon the extraction of their teeth and the insertion of a plate as true economy. How much greater benefit could they frequently receive by appropriating the money thus expended by attempting to preserve those few teeth which were defective at the time that they had them *all* extracted regardless of their condition.

The fourth ill effect, the increase in diseased conditions of the mouth and teeth. This result of itself should be a sufficient argument to cause every conscientious dentist to make an effort to overcome it by giving more thoughtful advice in regard to artificial work. Besides the inflamed condition found in the mucous membrane produced by the non-conductibility of the rubber, there is the more serious fault of the production of decay about the necks of the teeth owing to the ill adaptation and unnecessary thickness frequently found upon a rubber plate at this point.

Furthermore, the congested and inflamed condition of the mucous membrane causes additional trouble by producing an unhealthy vascular and nervous supply to the alveolar border, sooner or later causing rapid absorption of that process, and a consequent loosening of the remaining teeth. In the examination of fifty-three mouths, wearing either a partial or entire denture upon a plastic base, this condition was plainly discernible in forty-four of them, the remaining nine showing little or no detrimental result. So far as my observations have informed me, it requires from four to five years continual use of a rubber plate to produce the most disastrous effects, the most aggravated results appearing in those patients wearing their dentures while sleeping.

Having thus enumerated a number of unfavorable results produced from the misuse of rubber, together with the circumstances under which it may be advantageously used, it yet remains for me to briefly speak of the good and bad in metallic bases. First as to the metals to be used for this purpose. *In the proportion that gold is the best metal to be used in filling teeth, a corresponding preference should be given to its use in the construction of plates.*

Silver, while much to be preferred to rubber for small partial plates, can only boast of its cheapness as a further recommendation. The weight of aluminum is all that can be said in favor of that material. As I have previously admitted, while it is not possible to use gold in every case, it is certainly deserving of a more general use. It has for its advantages, conductibility, perfect adaptation, cleanliness, strength, and lack of bulk. In the fifty-three mouths previously referred to as wearing rubber plates, twenty-two of them were replaced with gold as a base. Every one of them soon began to exhibit a marked improvement, and those which it was possible to keep under observation resulted in a complete transformation, the inflamed, spongy mucous membrane having disappeared, while teeth that were much disturbed have taken on new life. What other changes might be brought about by a more generous use of this precious metal? Many of the ill results produced by the indiscriminate use of rubber would be overcome, mechanical dentistry would soon have a fascinating influence over the average practitioner, and the desire of divorcement of the two branches of our work would disappear, producing altogether a more generous and more useful profession.

To encourage a more general use of metallic bases, I have devised a method by which the swaging process is much simplified. The principle, while not entirely new, is nevertheless new in its application. By this method I dispense with the use of the horn mallet almost entirely, permitting a series of counter-dies (which I have named "progressive counter-dies") to perform the work previously accomplished with the horn mallet. The die is formed in the usual way, and the series of counter-dies are formed in the manner illustrated upon the black-board.

It may be argued that a plate swaged in this manner will not retain its shape so well as one coaxed into position by the use of the mallet. My idea is this, that the force of the blow being equally distributed to all portions of the plate, the result is the formation of a plate which will be much more rigid than one swaged in the old way. However, this may be a point for argument, and I hope to hear some remarks in reference to it.

No matter how great care is taken in swaging a plate with the horn mallet, every blow of the mallet is bound to be recorded upon the surface of the plate in the form of a bump, or a depression. These inequalities, while not necessarily visible to the eye after polishing the plate, may be plainly detected by the touch. The saving of time is a consideration. I have two plates here, one of them being swaged in seven minutes and the other in five. There being no scratches or imperfection in them, it only requires a few minutes' use of the brush-wheels to accomplish this part of the work.

